# Cerebrovascular insufficiency and amyloidogenic signaling in Ossabaw swine with cardiometabolic heart failure

**DOI:** 10.1172/jci.insight.143141

**Published:** 2021-05-24

**Authors:** Bradley J. Baranowski, Matti D. Allen, Jennifer N.K. Nyarko, R. Scott Rector, Jaume Padilla, Darrell D. Mousseau, Christoph D. Rau, Yibin Wang, M. Harold Laughlin, Craig A. Emter, Rebecca E.K. MacPherson, T. Dylan Olver

**Affiliations:** 1 Department of Health Sciences and; 2 Centre for Neuroscience, Brock University, St. Catharines, Ontario, Canada.; 3 Department of Physical Medicine and Rehabilitation, School of Medicine, Faculty of Health Sciences, Queen’s University, Kingston, Ontario, Canada.; 4 Department of Psychiatry, College of Medicine, University of Saskatchewan, Saskatoon, Saskatchewan, Canada.; 5 Department of Nutrition and Exercise Physiology, University of Missouri, Columbia, Missouri, USA.; 6 Research Service, Harry S. Truman Memorial Veterans’ Hospital, Columbia, Missouri, USA.; 7 Dalton Cardiovascular Research Center, University of Missouri, Columbia, Missouri, USA.; 8 Department of Genetics, School of Medicine, University of North Carolina at Chapel Hill, Chapel Hill, North Carolina, USA.; 9 Department of Anesthesiology, David Geffen School of Medicine, University of California, Los Angeles, Los Angeles, California, USA.; 10 Department of Biomedical Sciences, University of Missouri, Columbia, Missouri, USA.; 11 Department of Biomedical Sciences, Western College of Veterinary Medicine, University of Saskatchewan, Saskatoon, Saskatchewan, Canada.

**Keywords:** Cardiology, Neuroscience, Cardiovascular disease, Dementia, Heart failure

## Abstract

Individuals with heart failure (HF) frequently present with comorbidities, including obesity, insulin resistance, hypertension, and dyslipidemia. Many patients with HF experience cardiogenic dementia, yet the pathophysiology of this disease remains poorly understood. Using a swine model of cardiometabolic HF (Western diet+aortic banding; WD-AB), we tested the hypothesis that WD-AB would promote a multidementia phenotype involving cerebrovascular dysfunction alongside evidence of Alzheimer’s disease (AD) pathology. The results provide evidence of cerebrovascular insufficiency coupled with neuroinflammation and amyloidosis in swine with experimental cardiometabolic HF. Although cardiac ejection fraction was normal, indices of arterial compliance and cerebral blood flow were reduced, and cerebrovascular regulation was impaired in the WD-AB group. Cerebrovascular dysfunction occurred concomitantly with increased MAPK signaling and amyloidogenic processing (i.e., increased APP, BACE1, CTF, and Aβ40 in the prefrontal cortex and hippocampus) in the WD-AB group. Transcriptomic profiles of the stellate ganglia revealed the WD-AB group displayed an enrichment of gene networks associated with MAPK/ERK signaling, AD, frontotemporal dementia, and a number of behavioral phenotypes implicated in cognitive impairment. These provide potentially novel evidence from a swine model that cerebrovascular and neuronal pathologies likely both contribute to the dementia profile in a setting of cardiometabolic HF.

## Graphical abstract

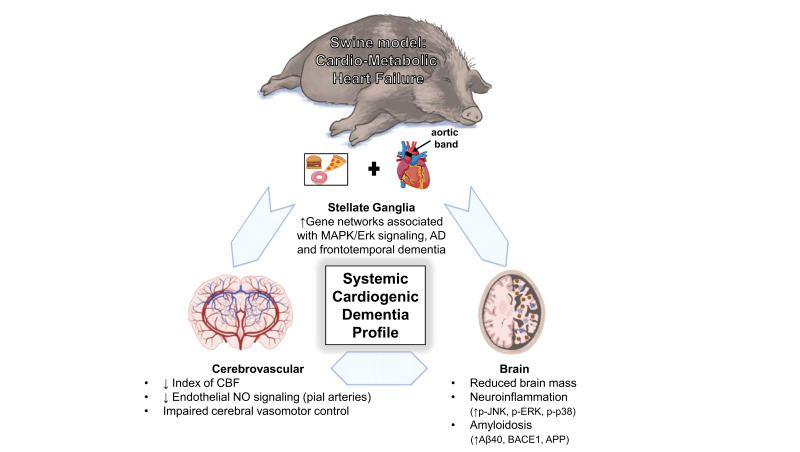

## Introduction

The term “cardiogenic dementia” was first introduced in the late 1970s to describe the link between cardiac and cognitive dysfunction (1). Estimates indicate up to 50% of patients with heart failure (HF) experience cardiogenic dementia (2, 3). Among patients with HF, cardiogenic dementia is associated with increased hospitalization, the loss of independence, and increased risk of mortality (4–7). Cardiogenic dementia is not characterized by a singular dementia phenotype and frequently includes aspects of vascular dementia as well as amyloidosis and Alzheimer’s disease (AD) (8). As with AD, cardiogenic dementia pathology appears to develop in the prefrontal cortex and hippocampus, regions involved closely in executive and memory function (8, 9).

Patients with HF with preserved ejection fraction (HFpEF; HF subtype reflecting ~50% of total HF cases) exhibit cardiogenic dementia, yet the etiology of disease remains unclear in this clinical population (2, 10–14). Current evidence indicates that HFpEF occurs more frequently in older women (versus men) (15) and reflects a total body syndrome affecting multiple organ systems and tissues beyond the heart, including the peripheral vasculature and the brain (8, 16). Mounting evidence suggests metabolic dysregulation and chronic low-grade inflammation represent a unifying link between cerebrovascular insufficiency and AD pathology (17, 18). Owing to structural and functional similarities between human and swine cardiovascular systems and brains (human and pig brains are gyrencephalic, containing >60% white matter) (19–21), swine models of disease may provide unique translational insight into multiorgan/system-level pathologies. Recently, our group characterized a large animal (e.g., swine) model of cardiometabolic HF (22) that was identified as a multihit model useful for the study of HFpEF by the National Heart, Lung, and Blood Institute/NIH HFpEF working group (23). This preclinical model exhibits key comorbidities of HF, including physical inactivity (decreased home-cage activity), obesity, dyslipidemia, insulin resistance, and elevated aortic systolic pressure. In addition, these animals display pathological features of HF, including lung congestion, systemic inflammation, concentric left ventricular remodeling, cardiac diastolic dysfunction, and preserved EF (22). Importantly, work from our lab demonstrates this model of HF induction impairs working memory performance (24), possibly in part through deficits in spatial learning or navigation (25). This model of HF induction was highlighted in the American Heart Association Scientific Statement for its utility as a model to better understand the pathogenesis of hypertensive cerebral damage (23). Using this disease platform to gain further insight into the heart-brain axis in HF, the purpose of the current study was to test the hypothesis that swine with cardiometabolic HF would exhibit a complex dementia phenotype involving cerebrovascular dysfunction as well as indices of neuroinflammation and AD-like amyloidosis. Specifically, we investigated indices of cerebral blood flow control in vivo as well as pial and brain parenchymal artery vasomotor control in isolated cerebral arterioles ex vivo. Further, because it interfaces with the heart-brain axis (26, 27), we examined the transcriptomic profile in the stellate ganglia. Last, given the established role of aberrant mitogenic signaling in AD pathology (28–31), transcriptomic profiling of the stellate ganglia was coupled with examination of MAPK pathway activation as well as key regulators of the generation of the β-amyloid peptide in the prefrontal cortex and hippocampus. The results of this study demonstrate swine with cardiometabolic HF displayed a combination of cerebrovascular and neuronal pathologies, indicating swine models of multiorgan disease may provide an excellent model to interrogate cardiogenic dementia.

## Results

### Physical characteristics, cardiovascular parameters, and cerebrovascular function.

Results describing physical characteristics as well as a cardiac, peripheral vascular, immune, and hepatic phenotype for the same animals used in this study are only summarized here as they were reported previously (22). Swine in the Western diet aortic banded (WD-AB) group were obese and dyslipidemic, exhibited steatohepatitis, and displayed evidence of HF, including pulmonary congestion, concentric left ventricle remodeling, diastolic dysfunction, impaired coronary microvascular vasomotor control, and genetic signatures consistent with an HF phenotype alongside normal EF (>45%). Additionally, swine in the WD-AB group displayed significantly less daily cage activity. Our previous work provides a detailed description of the multihit phenotype in this swine model and clearly establishes these characteristics of the model (22). Systolic blood pressure and pulse pressure were increased in the WD-AB group (*P* < 0.05; Supplemental Figure 1; supplemental material available online with this article; https://doi.org/10.1172/jci.insight.143141DS1).

**Figure 1:**
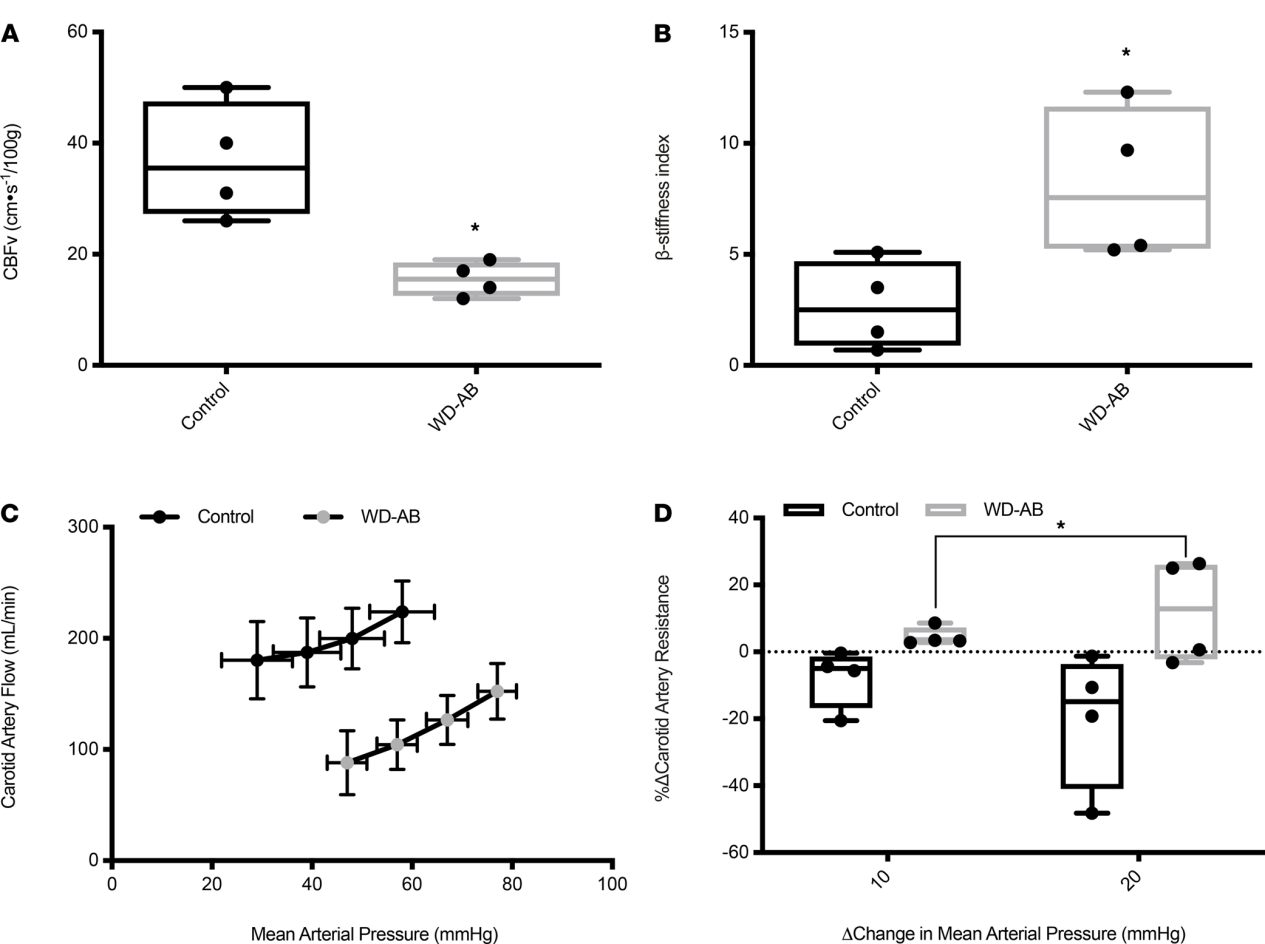
Baseline hemodynamics. (**A**) Cerebral blow flow velocity (CBFv); (**B**) β-stiffness index; (**C**) carotid artery flow during vena cava occlusion; (**D**) percentage change in carotid artery vascular resistance in response to a change in mean arterial pressure. Data analyzed using an unpaired, 2-tailed *t* test and a 2-way ANOVA. Values are represented as mean ± SEM. The box plots depict the minimum and maximum values (whiskers), the upper and lower quartiles, and the median. The length of the box represents the interquartile range. Significance indicated by *P < 0.05 compared with control. (CON; *n* = 5, WD-AB; *n* = 4.)

Brain mass was lower in the WD-AB group (control = 110 ± 3 vs. WD-AB = 93 ± 2 g, *P* < 0.01). Cerebral blood flow velocity (absolute and scaled to brain mass) was lower and β-stiffness index was greater in the WD-AB group (*P* < 0.01; Figure 1, A and B). Baseline carotid artery blood flow was lower and carotid artery vascular resistance was greater in the WD-AB group (carotid artery flow: control = 315 ± 145 vs. WD-AB = 169 ± 26 mL/min, *P* < 0.05; carotid artery vascular resistance: control = 0.23 ± 06 vs. WD-AB=0.54 ± 0.08 mmHg/mL/min, *P* < 0.05). Absolute data for carotid artery blood flow during central hypovolemia are presented in Figure 1C. The percentage change in carotid artery vascular resistance during central hypovolemia was both positive (i.e., increased) and greater in the WD-AB group (*P* < 0.01; Figure 1D).

**Figure 2:**
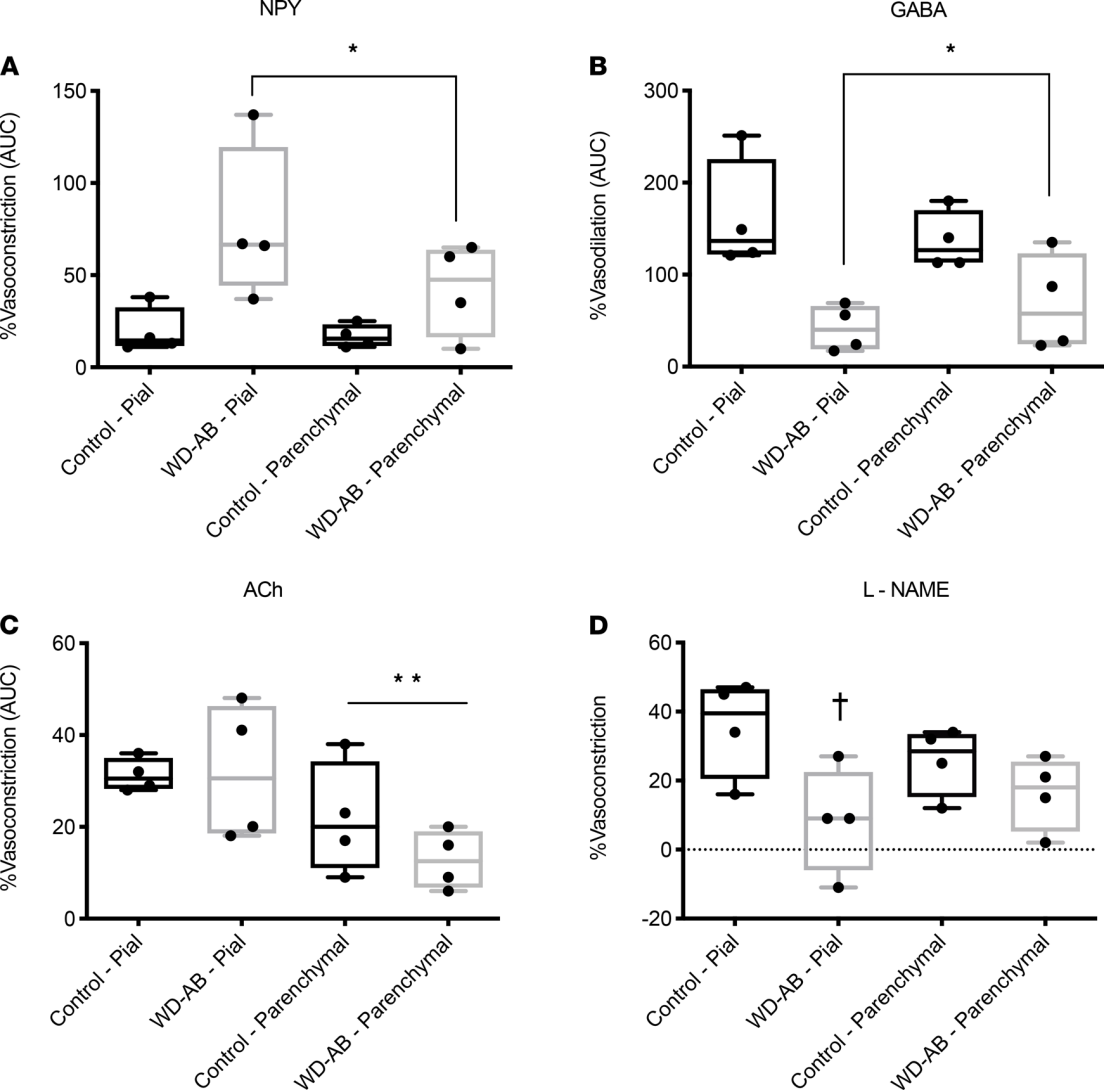
Vasomotor control. (**A**) NPY-induced %vasoconstriction (AUC); (**B**) GABA-induced %vasodilation (AUC); (**C**) ACh-induced %vasoconstriction (AUC); (**D**) l-NAME–induced %vasoconstriction. Data analyzed using a 2-way ANOVA. Values are represented as mean ± SEM. The box plots depict the minimum and maximum values (whiskers), the upper and lower quartiles, and the median. The length of the box represents the interquartile range. Main effect indicated by *P < 0.05 compared with control, **P < 0.05 compared with pial artery; interaction effect indicated by †*P* < 0.05 compared with control pial. (CON; *n*= 5, WD-AB; *n* = 4.)

In isolated pial and parenchymal arteries, maximal luminal diameters were similar between groups and artery types (control pial = 284 ± 48 vs. WD-AB pial = 262 ± 36 vs. control parenchymal = 177 ± 11 vs. WD-AB parenchymal = 294 ± 70 μm; *P* ≥ 0.32). The wall-to-lumen ratio was similar between groups but was greater in pial versus parenchymal arteries (control pial = 0.21 ± 0.03 vs. WD-AB pial = 0.23 ± 0.01 vs. control parenchymal = 0.16 ± 0.01 vs. WD-AB parenchymal = 0.18 ± 0.01; *P* < 0.01). The net vasoconstriction in response to neuropeptide Y (NPY) treatment was greater and the net vasodilation in response to γ-aminobutyric acid (GABA) treatment was attenuated in the WD-AB group (main effect of WD-AB; *P* < 0.05; Figure 2, A and B). The net vasoconstriction in response to acetylcholine (ACh) treatment was similar between groups (*P* = 0.31) but was lower in parenchymal versus pial arteries (main effect of artery type; *P* = 0.01; Figure 2C). The vasoconstrictor response following treatment with the nitric oxide synthase inhibitor nitro-l-arginine methyl ester (l-NAME) was reduced in WD-AB pial arteries (*P* < 0.05; Figure 2D).

### Transcriptomic profile.

**Figure 3:**
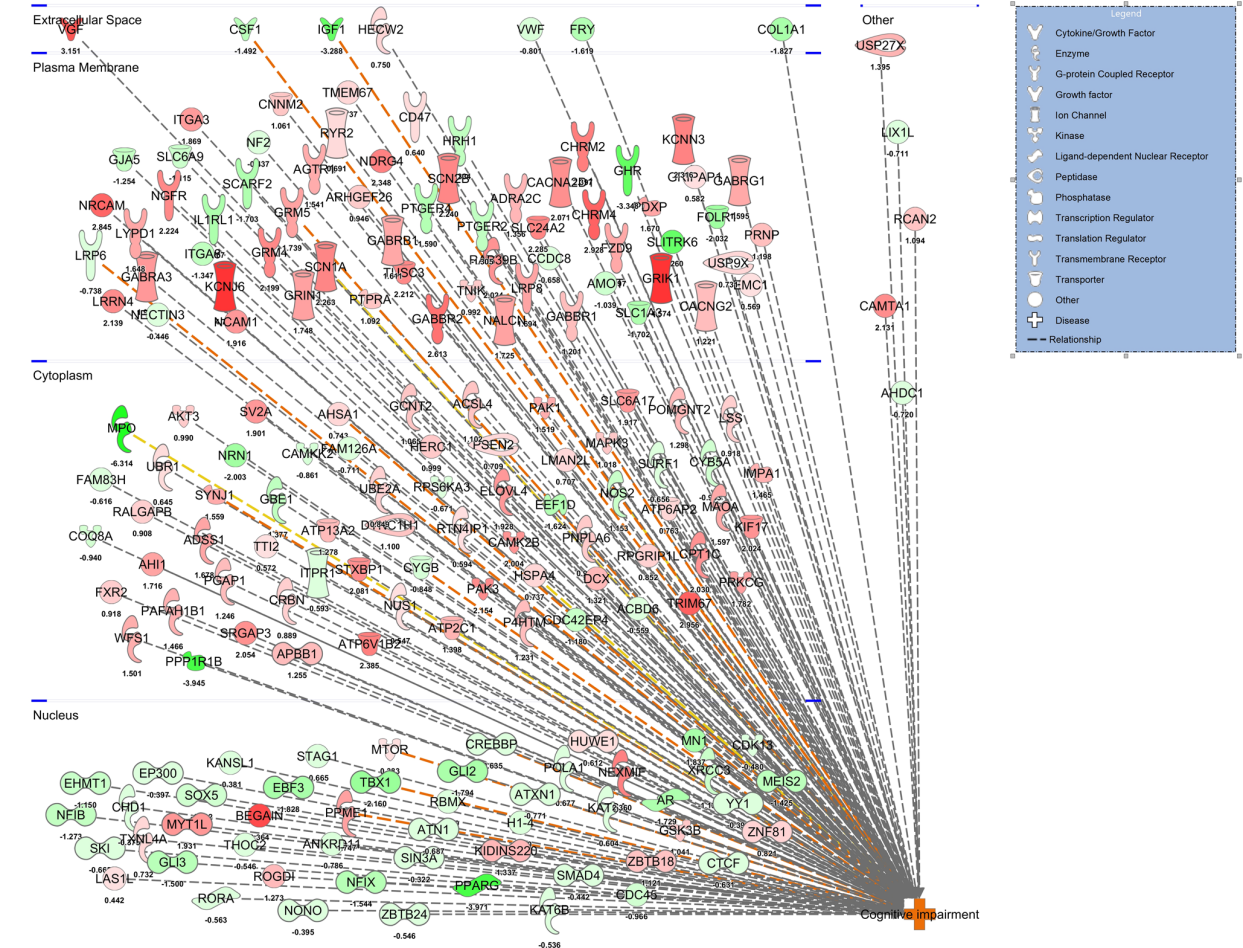
Gene Ontology and Ingenuity Pathway Analysis of induced gene pathways in the stellate ganglia that are differentially expressed between the control and WD-AB group. Unbiased Ingenuity Pathway Analysis depicting significant gene interactions in the WD-AB group that are associated with cognitive impairment. (CON; *n* = 5, WD-AB; *n* = 4.)

The stellate ganglia transcriptome signature was compared between control and WD-AB groups. Based on Gene Ontology analysis that identified differentially expressed genes, the molecular signatures in the stellate ganglia were associated with those present in the cerebral cortex and hippocampus (Table 1; *P* < 0.001). Furthermore, there was an enrichment of gene networks associated with AD, frontotemporal dementia, stroke, MAPK/ERK signaling pathways, and a number of phenotypes implicated in cognitive impairment (Table 1; *P* < 0.001). Unbiased Ingenuity Pathway Analysis (QIAGEN) revealed a cluster of significant gene interactions in the WD-AB group were associated with cognitive impairment (Figure 3; *P* < 0.001). For a complete list of matched genes, please see Supplemental Table 1.

### Molecular markers of neuroinflammation and AD.

**Figure 4:**
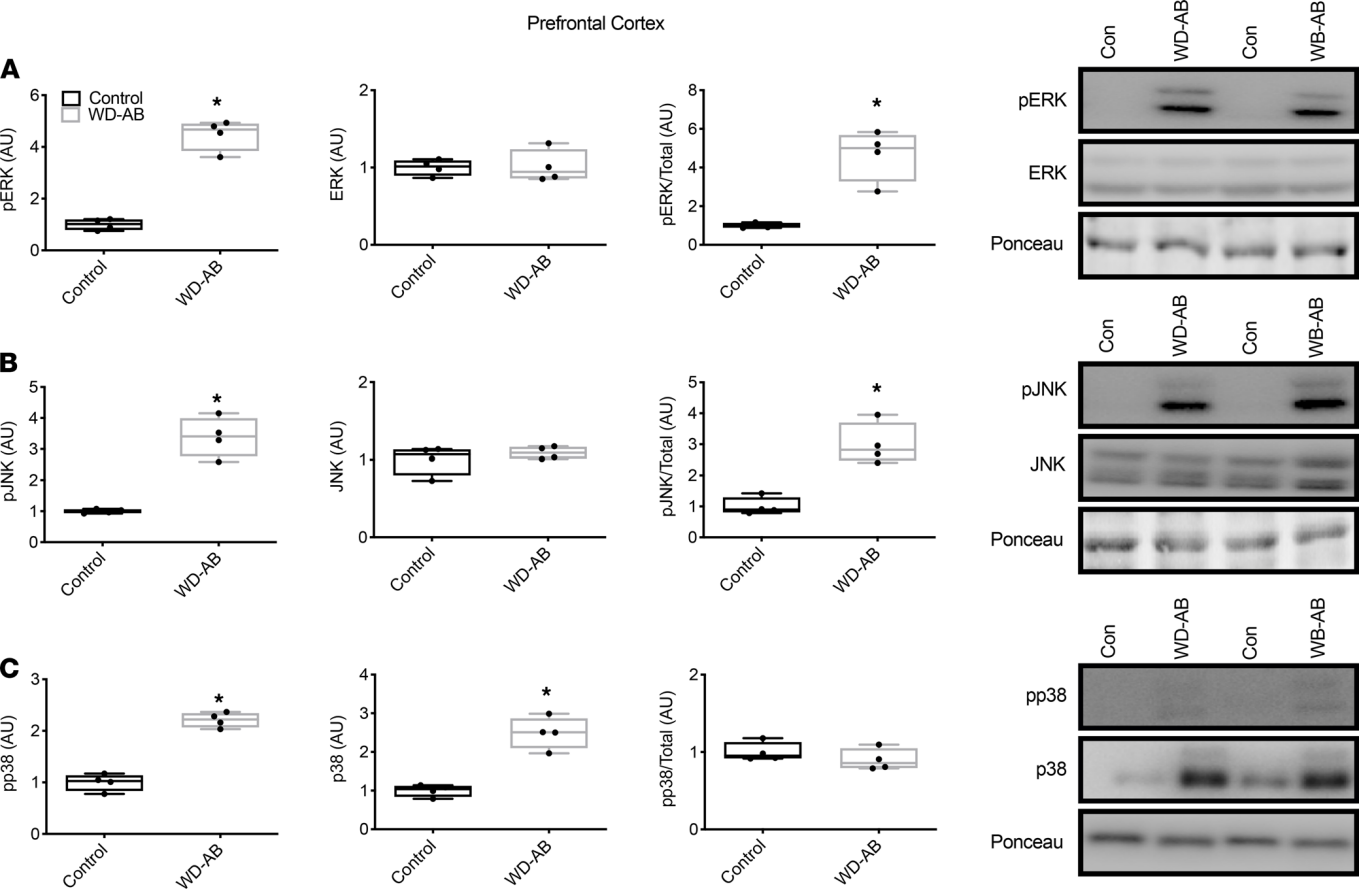
MAPKs in prefrontal cortex. (**A**) Phosphorylated ERK (p-ERK), total ERK, and the ratio of p-ERK/total ERK in the prefrontal cortex as well as a representative blot; (**B**) p-JNK, total JNK, and the ratio of p-JNK/total JNK in the prefrontal cortex as well as a representative blot; (**C**) p-p38, total p38, and the ratio of p-p38/total p38 in the prefrontal cortex as well as a representative blot. Data analyzed using an unpaired, 2-tailed *t* test. Values are represented as mean ± SEM. The box plots depict the minimum and maximum values (whiskers), the upper and lower quartiles, and the median. The length of the box represents the interquartile range. Significance indicated by *P < 0.05 compared with control. (CON; *n* = 5, WD-AB; *n* = 4.)

**Figure 5:**
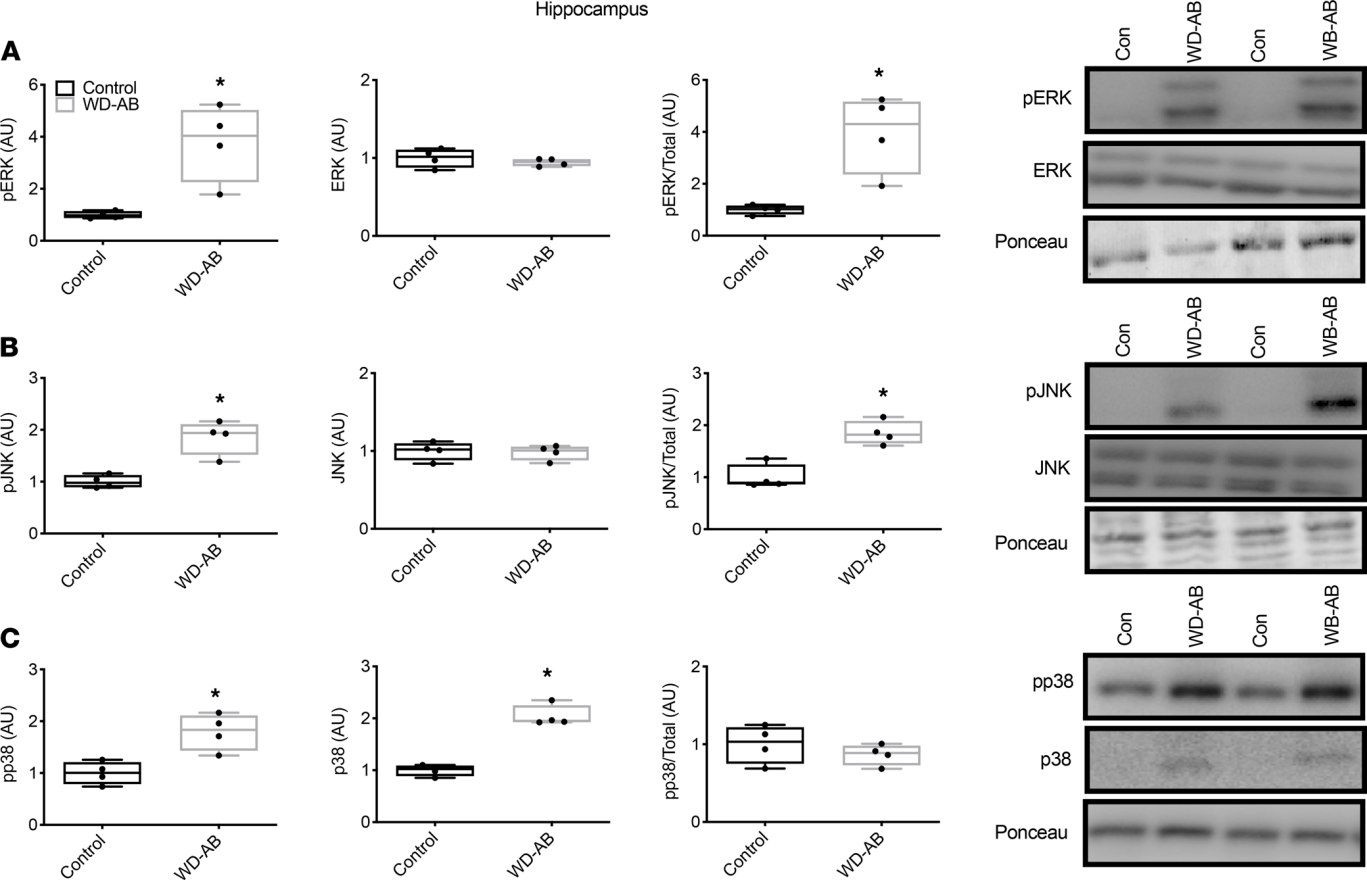
MAPKs in hippocampus. (**A**) p-ERK, total ERK, and the ratio of p-ERK/total ERK in the hippocampus as well as a representative blot; (**B**) p-JNK, total JNK, and the ratio of p-JNK/total JNK in the hippocampus as well as a representative blot; (**C**) p-p38, total p38, and the ratio of p-p38/total p38 in the hippocampus as well as a representative blot. Data analyzed using an unpaired, 2-tailed *t* test. Values are represented as mean ± SEM. The box plots depict the minimum and maximum values (whiskers), the upper and lower quartiles, and the median. The length of the box represents the interquartile range. Significance indicated by *P < 0.05 compared with control. (CON; *n* = 5, WD-AB; *n* = 4.)

**Figure 6:**
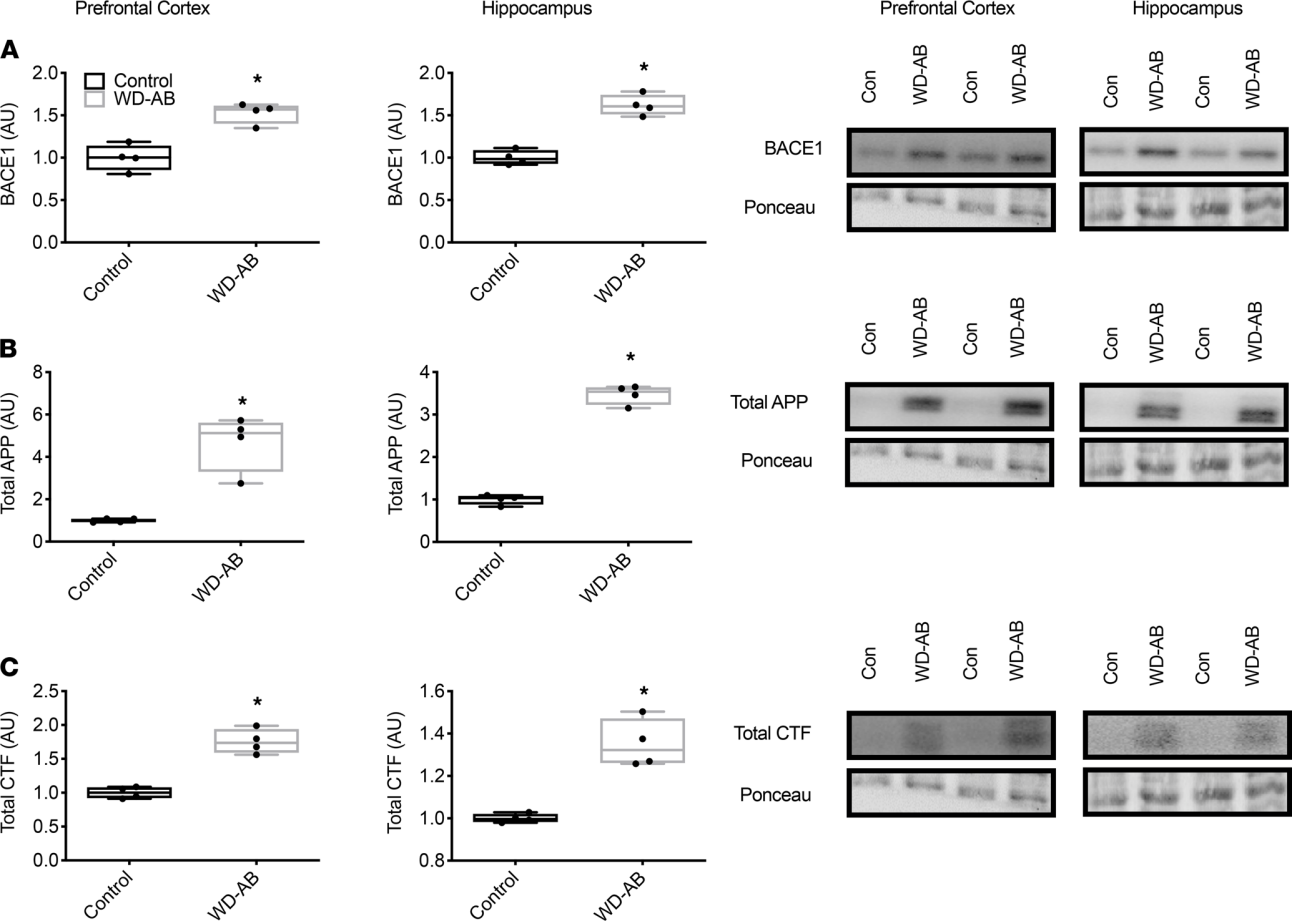
Western blot markers for the amyloidogenic cascade. (**A**) BACE1 in the prefrontal cortex and hippocampus as well as representative blots; (**B**) total APP in the prefrontal cortex and hippocampus as well as representative blots; (**C**) CTF in the prefrontal cortex and hippocampus as well as representative blots. Data analyzed using an unpaired, 2-tailed *t* test. Values are represented as mean ± SEM. The box plots depict the minimum and maximum values (whiskers), the upper and lower quartiles, and the median. The length of the box represents the interquartile range. Significance indicated by *P < 0.05 compared with control. (CON; *n* = 5, WD-AB; *n* = 4.)

**Figure 7:**
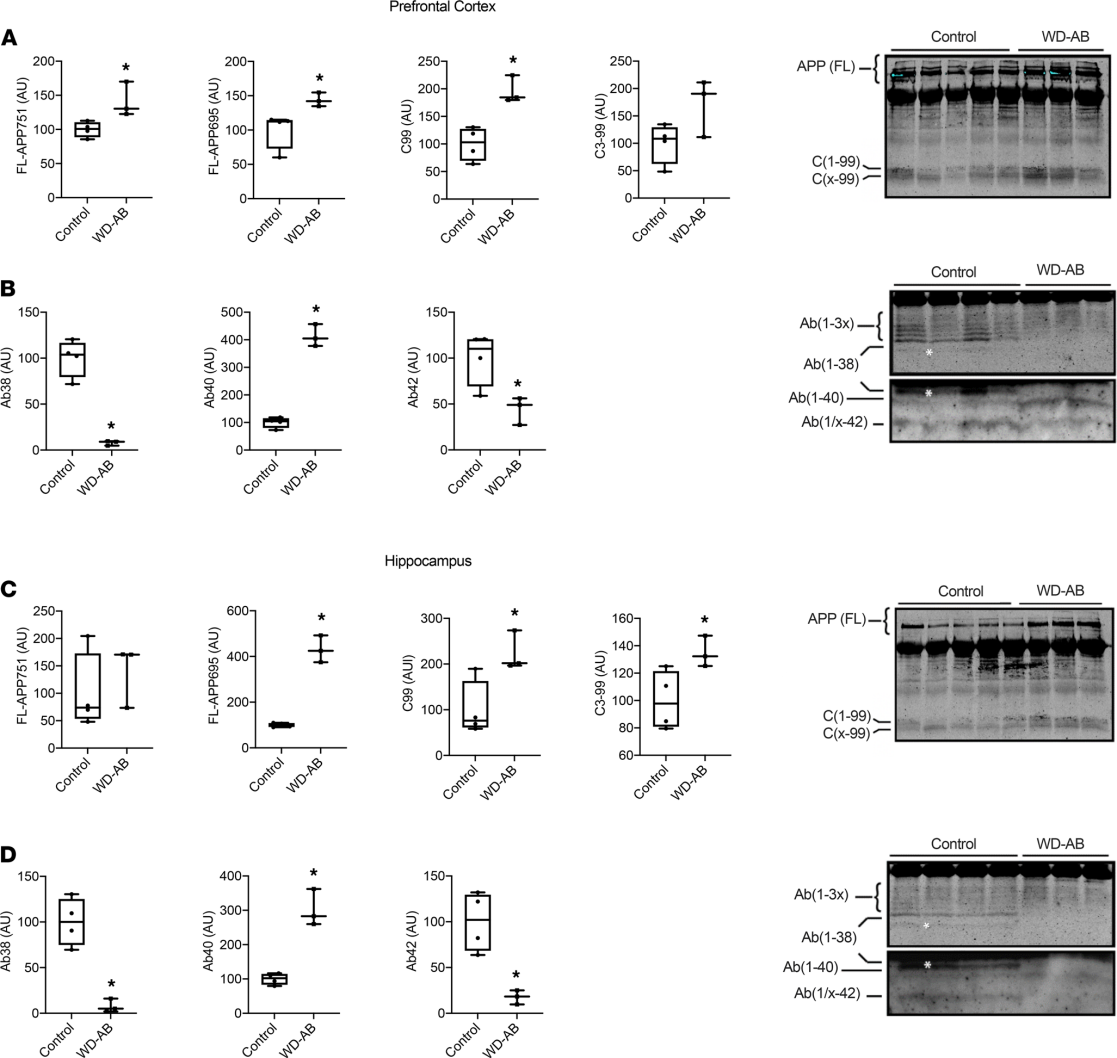
Immunoprecipitation for markers of the amyloidogenic cascade. (**A**) FL-APP751, FL-APP695, C99, and C3-99 in the prefrontal cortex as well as representative blots for FL-APP and CTF; (**B**) Aβ38, Aβ40, and Aβ42 in the prefrontal cortex as well as representative blots for Aβ species; (**C**) FL-APP751, FL-APP695, C99, and C3-99 in the hippocampus as well as representative blots for FL-APP; (**D**) Aβ38, Aβ40, and Aβ42 in the hippocampus as well as representative blots for Aβ species. Data analyzed using an unpaired, 2-tailed *t* test. Values are represented as mean ± SEM. The box plots depict the minimum and maximum values (whiskers), the upper and lower quartiles, and the median. The length of the box represents the interquartile range. Significance indicated by *P < 0.05 compared with control. (CON; *n* = 5, WD-AB; *n* = 4.)

Phosphorylated ERK and JNK and the ratio of phosphorylated to total ERK and JNK were elevated in the WD-AB group (*P* < 0.01), in the absence of differences in total ERK and JNK in the prefrontal cortex (*P* ≥ 0.42) (Figure 4, A and B), whereas both phosphorylated and total p38 were greater in the WD-AB group in the prefrontal cortex (Figure 4C; *P* < 0.01). Similar to the prefrontal cortex, phosphorylated ERK and JNK and the ratios of phosphorylated to total ERK and JNK were greater in the WD-AB group (*P* < 0.01), and total ERK and JNK were similar between groups in the hippocampus (*P* ≥ 0.43) (Figure 5, A and B). Phosphorylated and total p38 protein content were also upregulated in the WD-AB group in the hippocampus (*P* < 0.01) (*P* ≥ 0.37; Figure 5C). Levels of beta-secretase 1 (BACE1), amyloid precursor protein (APP), and BACE1-mediated APP–C-terminal fragment (APP-CTF) were all greater in the WD-AB group in both the prefrontal cortex (*P* < 0.01) and the hippocampus (*P* < 0.01; Figure 6, A–C). Immunoprecipitation for specific splice variants of AD-related amyloidosis revealed that full-length APP splice variants APP751 and APP695 were greater in the prefrontal cortex in the WD-AB group (*P* ≤ 0.05; Figure 7A). Levels of the APP-CTF and C99 fragment were greater (*P* < 0.01; Figure 7A), while increases in the levels of C3-99 fragment approached significance in the prefrontal cortex of the WD-AB group (*P* = 0.09; Figure 7A). The levels of the Aβ40 peptide were greater in the prefrontal cortex of the WD-AB group (*P* ≤ 0.01), whereas levels of the Aβ38 and Aβ42 peptides were lower in these same extracts (*P* < 0.05; Figure 7B). In contrast, differences in hippocampal levels of APP751 were not significant between groups (*P* = 0.47), while the levels of APP695 were greater in the WD-AB group (*P* < 0.01; Figure 7C). With respect to the APP-CTF, both C99 and C3-99 fragments were higher in the hippocampus of WD-AB swine (*P* ≤ 0.05; Figure 7C). Similar to the prefrontal cortex, the levels of the Aβ40 peptide were greater in the hippocampus, but levels of the Aβ38 and Aβ42 peptides were lower in the WD-AB group (*P* < 0.01; Figure 7D).

## Discussion

The results of the current investigation suggest that the development of cerebrovascular dysfunction is coupled with neuroinflammation and amyloidosis in this swine model of multihit cardiometabolic HF (see Graphical abstract). Specifically, the data reveal that indices of carotid arterial compliance and CBF were reduced, and cerebrovascular regulation was altered in the WD-AB group. Cerebrovascular dysfunction was coupled with increased neuronal ERK, JNK, and p38 signaling as well as amyloidogenic processing indicated by increased APP, BACE1, CTF, and Aβ40 content in the prefrontal cortex and hippocampus of WD-AB swine. The transcriptome signature in the stellate ganglia revealed there was an enrichment of gene networks associated with disease and behavioral phenotypes consistent with dementia. Collectively, these data support the notion that cardiometabolic HF reflects a total body syndrome affecting both cardiac and extracardiac tissues. To our knowledge these data represent the first evidence from a swine model that cerebrovascular and neuronal pathologies develop concomitantly in the setting of experimental cardiometabolic HF.

Despite normal resting EF% values in this HF model, the index of CBF was decreased, and cranial vascular resistance was increased during central hypovolemia in WD-AB swine. We previously reported indices of cerebral perfusion are correlated with memory performance in intact (male and female) and ovariectomized (female) pigs with and without AB, highlighting the potential risk of cerebral insufficiency in HF (24, 25). In HF patients with impaired systolic function (HF with reduced EF), reduced cardiac output and EF% are believed to be primary contributors to cerebral hypoperfusion and dysregulation (6, 32–34). However, in HFpEF and this preclinical model of cardiometabolic HF, cardiac output and EF% are normal under resting conditions (35, 36). Combined with the current data, this raises the possibility that cerebrovascular dysfunction develops independent of resting systolic impairment in this HF population. In isolated cerebral arteries from the WD-AB group, we also found decreased vasodilation in response to GABA and increased vasoconstriction in response to NPY, supporting the interpretation of cerebrovascular dysfunction in this model. GABA and NPY are vasoactive neurotransmitters that can stimulate both dilation and constriction depending on concentration and location of signaling (i.e., vascular smooth muscle or endothelium) (37–39). Thus, altered vasoreactivity to GABA and NPY may contribute to impairments in cerebrovascular regulation. Whether disturbed cerebrovascular control is a key contributor to the cardiogenic dementia profile in HF warrants further investigation.

We also found that inhibition of NO synthase (NOS) caused greater pial artery vasoconstriction in control than WD-AB swine, suggesting basal endothelial NO signaling is decreased in the WD-AB group. Evidence indicates that pial arteries contribute significantly to cerebrovascular resistance (40), and although NO is not implicated in vasoreactivity to GABA (37), inhibition of endothelial NOS (eNOS) attenuates NPY-induced vasodilation (38). Furthermore, inhibition of eNOS can decrease basal CBF (41–44). Thus, impaired endothelial NO signaling in pial arteries may represent an additional mechanism that contributes to impaired cerebrovascular regulation in the WD-AB group. Earlier experiments revealed WD-AB swine display impaired cerebral artery vasodilation in response to insulin and sodium nitroprusside, both of which function through an NO-dependent pathway (22). Further, our previous work in female swine reveals AB, in the absence of WD, decreases cerebral eNOS protein content (25). Collectively, the data highlight endothelial dependent and independent impairments in NO signaling may be involved in reduced CBF and impaired cerebrovascular regulation in experimental HF. Whereas impaired NO signaling is considered a hallmark feature of HFpEF (45–49), the notion that it contributes to cardiogenic dementia in this population has not been validated clinically in humans.

In addition to impaired NO signaling, amyloidosis may be involved in neurodegeneration in HFpEF (8, 17, 18, 50, 51). In the present study, the WD-AB group displayed amyloidosis in the brain, evidenced by increased APP, BACE1, and CTF content as well as increased accumulation of Aβ40 peptides. Amyloidosis, which is implicated in cerebral amyloid angiopathy and AD (52, 53), is reflected by aberrant cleavage of the full-length APP by BACE1 resulting in the production of soluble APP fragment and a membrane-bound CTF (54). The membrane-bound CTF is subsequently cleaved by γ-secretase, releasing it from the membrane and resulting in the production of extracellular Aβ peptides that vary in length between 38 and 43 amino acid chains (52, 53). Of note, Aβ40 and Aβ42 peptides can accumulate in neuronal or vascular cells within the brain and promote cellular dysfunction and cell death as well as gross neurological impairments, such as mild cognitive impairment or dementia (55). Recently, a study revealed Aβ pathology, and notably accumulation of Aβ40, affects the brain (and heart) in patients with combined cardiac diastolic dysfunction and AD (51). Importantly, NO is a negative upstream regulator of APP and BACE1 (56–59). Pharmacological inhibition of eNOS as well as its second messenger cyclic guanosine monophosphate results in increased APP and BACE1 content in human brain microvascular endothelial cells (56). Moreover, eNOS heterogeneous and homogenous knockout mice display increased Aβ40 content in the brain (56, 57). With respect to the current findings, these data implicate a potential mechanism linking metabolic dysregulation with impaired NO signaling and ensuing amyloidosis in the setting of experimental cardiometabolic HF.

Beyond impaired NO signaling, increased neuroinflammation, reflected by increased ERK and JNK signaling as well as p38 content, may have also contributed to amyloidosis in the WD-AB group. The activation of the MAPK pathway, specifically ERK, JNK, and p38, can result in an upregulation of BACE1 expression and content (60, 61), as well as direct phosphorylation of APP (62, 63), rendering it more susceptible to BACE1 cleavage. Further, these MAPKs exacerbate neuronal apoptosis, leading to increased brain atrophy and cognitive impairments (64, 65). Indeed, the WD-AB swine displayed an enhanced MAPK pathway activation concurrently with a reduction in brain mass (wet weight), indicative of neurodegeneration. With respect to the heart-brain axis (26, 27), Gene Ontology analysis of the stellate ganglia revealed an enrichment of several gene signatures associated with MAPK/ERK signaling and AD, highlighting the systemic nature of this disease phenotype. Given that brain samples from patients with AD and transgenic rodent models display higher levels of phosphorylated JNK, ERK, and p38 (66–68), and cardiac transcriptomic data from patients with HFpEF display enhanced MAPK signaling as well as an enrichment in gene networks associated with AD (69), it is conceivable that MAPK pathway activation contributes to the cardiogenic dementia profile.

Despite evidence of increased MAPK pathway activation and amyloidogenic signaling (i.e., increased APP, BACE1, CTF, and Aβ40) in WD-AB swine, Aβ lengths 42 and 38 were lower in the WD-AB group. These findings suggest that increased APP processing in WD-AB is associated with an apparent shift in the preferential cleavage of the CTF to produce Aβ40. This is not unexpected given that an increase in BACE1 (as we have demonstrated herein) is known to preferentially generate the Aβ40 peptide (70). In keeping with our model, accumulation of the Aβ40 peptide is associated more so with cerebral amyloid angiopathy (71) rather than with a pathologic diagnosis of pure AD, which is more closely associated with an accumulation of the aggregation-prone and neurotoxic Aβ42 peptide (72). However, in regard to cardiogenic dementia and cardiovascular disease, emerging evidence supports a strong link to the Aβ40 species, which also matches the current findings (17, 18, 73, 74). For example, while eNOS heterogeneous and homogenous knockout mice display increased Aβ40 content in the brain, Aβ42 is not detectable in the brains of these mice (56, 57). In addition, in patients with combined diastology and AD, while both Aβ40 and Aβ42 are increased, the accumulation of Aβ40 in the brain appears greater (51). Of note, evidence indicates that Aβ40, but not Aβ42, elicits cerebral vasoconstriction (75–77). When considered with the current data, these findings raise the question of the role of different Aβ species in varying disease states, as Aβ40 may be a critical feature of the dementia profile in this model of cardiometabolic HF.

### Study limitations.

The current data provide insight regarding the development of cerebrovascular and neuronal pathologies in a swine model of experimental cardiometabolic HF. However, owing to the lack of nutritional control groups, the contribution from the WD or individual components of the WD (i.e., cholesterol, saturated fat, sugar, or total kilocalories) could not be determined. Further, although the molecular data are in close alignment with brain biopsy data from AD patients with diastology (51) as well as cardiac biopsy data from HFpEF patients (78), the lack of histopathological and behavioral data herein limit the interpretation of the present results. Although our earlier work demonstrates AB alone and in conjunction with ovariectomy (experimental menopause) induces impairments in working memory, future work is warranted to assess the contribution of singular comorbidities (i.e., obesity, hypertension, etc.) or dietary components (i.e., cholesterol, sugar, etc.) on cerebrovascular and brain function in HF.

### Perspectives.

Mounting evidence indicates that extracardiac vascular and neuronal pathologies may contribute to the dementia profile in the setting of HF and cardiovascular disease (8, 9, 17, 18). Hypertension, large artery stiffening, cerebrovascular dysfunction, neuroinflammation, and amyloidosis are all extracardiac indicators that may accompany or precede dementia in the setting of HF. The development of translational animal models is necessary to better understand the pathophysiology of multiorgan diseases. This study provides evidence that extracardiac vascular and neuronal pathologies develop concurrently and independent of resting cardiac systolic impairment in this swine model of multihit cardiometabolic HF. Furthermore, in line with clinical observations (8, 9), neuronal maladaptation, herein reported as increased MAPK pathway activation coupled with increased APP processing, develop in both the prefrontal cortex and hippocampus. These data support the concept that cardiometabolic HF phenotypes reflect a total body syndrome affecting extracardiac organs, including the cerebrovasculature and the brain (8, 16–18), and that the cardiogenic dementia profile is not always characterized by a singular dementia phenotype (8). Given there is no effective intervention for cardiogenic dementia, it is important to determine what significant vascular and neurological changes precede and contribute to the development of cognitive decline in HF, toward identifying targets for early diagnosis and disease prevention.

## Methods

### Experimental design.

Data from these same animals and details of this model were originally described (22). Intact female Ossabaw swine (15–20 kg; 2 months old) were divided randomly into 2 groups: nonsham sedentary control (CON; *n* = 5) and WD-AB (*n* = 4). Female pigs were used because HFpEF, a form of cardiometabolic HF, disproportionally affects older women (~2:1 vs. men) (15).

The CON group ingested a standard chow diet (5L80, LabDiet; 3.03 kcal/g, carbohydrate = 71%, protein = 18.5%, and fat = 10.5%; 500 g/d), and the WD-AB group ingested a WD (1000 g/d) high in fat, high fructose corn syrup, and cholesterol (5B4L, LabDiet; 4.14 kcal/g; carbohydrate = 40.8% [17.8% of total calories from high fructose corn syrup], protein = 16.2%, fat = 43%, 2% cholesterol wt/wt) (22, 79). At 6 months of age, swine in the WD-AB group underwent aortic banding procedures to induce HF as previously described (24, 25, 80). Briefly, under anesthesia, a trans-stenotic systolic gradient of approximately 70 mmHg (72 ± 2 mmHg) was achieved using phenylephrine (i.v. 1–3 μg/kg/min) to maintain a distal peripheral vascular mean arterial pressure and heart rate at approximately 90 mmHg (87 ± 2 mmHg) and 85 beats/min (84 ± 3), respectively. At euthanasia, pial and brain parenchymal arteries were harvested for isolated arterial experiments, and the hippocampus as well as the prefrontal cortex were harvested for molecular experiments (i.e., immunoprecipitation and immunoblotting). In addition, the stellate ganglia were harvested for RNA sequencing (RNA-Seq).

### RNA-Seq, principal component analysis, weighted gene coexpression network analysis, module enrichment analysis, and Ingenuity Pathway Analysis.

As previously described (81–83), total RNA was isolated using TRIzol (Thermo Fisher Scientific), Illumina protocol was used to prepare (75 bp paired-end) RNA-Seq libraries, and libraries were sequenced on 1 lane on the Illumina Genome Analyzer II platform. Transcriptomic expression was identified using the Salmon 0.8 algorithm (84) using the seqBias and gcBias flags, and principal component analysis on all expressed and varying genes (fragments per kilobase million [FPKM] > 1, coefficient of variation > 5%) using the prcomp R function and the ggbiplot R package (85) was used to identify and remove outliers. All genes with average FPKM ≥ 1 across all samples were analyzed using the weighted gene coexpression network analysis (WGCNA) algorithm using default parameters from the provided tutorials (86), except where noted below (85). Briefly, Pearson correlations were determined for each pair of expressed transcripts. Thereafter, correlations were converted into an approximately scale-free correlation matrix by applying a factor of the power of 6 to each correlation. Adjusted correlations were transformed into a Topological Overlap Matrix (TOM) using the following equation:

  Equation 1
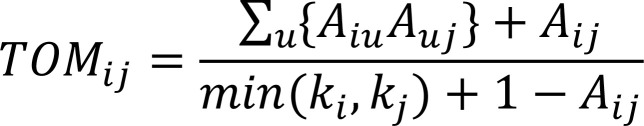


where *i* and *j* are the pair of genes to be analyzed, *u* is the set of all other genes, *A* is the adjusted correlation matrix, and *k* is the degree of the node. TOM scores were subsequently transformed to DistTOM scores by subtracting TOM from 1. The dynamic tree cut algorithm was applied on the DistTOM matrix to identify modules, and the first principal component of the genes in each module was used to determine eigengenes. Modules whose eigengenes had a Pearson correlation of greater than 0.8 were combined for downstream analyses. As described previously (87), correlations were then visualized using the heatmap function in WGCNA. GeneAnalytics was used for Model Enrichment (88) to identify enriched biological categories. Significance was determined by a Benjamini-Hochberg–corrected binomial test reported at the *P* < 0.05 level. Using Ingenuity Pathway Analysis, networks were generated based on the list of differentially expressed genes and subjected to statistical analysis as described previously (22, 89, 90).

### Statistics.

Brain mass, CBF velocity, carotid artery blood flow, carotid artery vascular resistance, blood pressure, and β-stiffness index were analyzed using a 2-tailed, unpaired *t* test. Percentage change (%) in cranial vascular resistance (at 10 and 20 mmHg reductions in mean arterial pressure) during central hypovolemia was analyzed using a mixed-model repeated measures ANOVA (group × mean arterial pressure). Similarly, vasomotor control was analyzed using a mixed-model repeated measures ANOVA (group × artery type). Significant interactions were explored using a post hoc Student-Newman-Keuls test. Molecular markers of neuroinflammation and AD in the prefrontal cortex and hippocampus were analyzed using an unpaired, 2-tailed *t* test. Where possible individual data are presented, and all data are presented as mean ± SEM. Significance is reported at the *P* ≤ 0.05 level. Molecular analyses of brain samples from 1 control animal revealed they were a statistical outlier, defined as values > 2 SD for >10 outcome measures. Therefore, this control sample was removed from the study. Last, owing to limited tissue sample, immunoprecipitation experiments were only conducted on 3 animals from the WD-AB group. Where possible, all individual data are plotted. See Supplemental Methods for a comprehensive description of experimental methods used in the current study.

### Study approval.

All animal protocols were in accordance with the “U.S. Government Principles for the Utilization and Care of Vertebrate Animals Used in Testing Research and Training” and approved by the University of Missouri Animal Care and Use Committee.

## Author contributions

BJB, MDA, JNKN, RSR, JP, DDM, CDR, YW, MHL, CAE, REKM, and TDO contributed to data collection and analyses. BJB and TDO composed the manuscript. BJB, MDA, JNKN, RSR, JP, DDM, CDR, YW, MHL, CAE, REKM, and TDO edited the manuscript.

## Supplementary Material

Supplemental data

## Figures and Tables

**Table 1:**
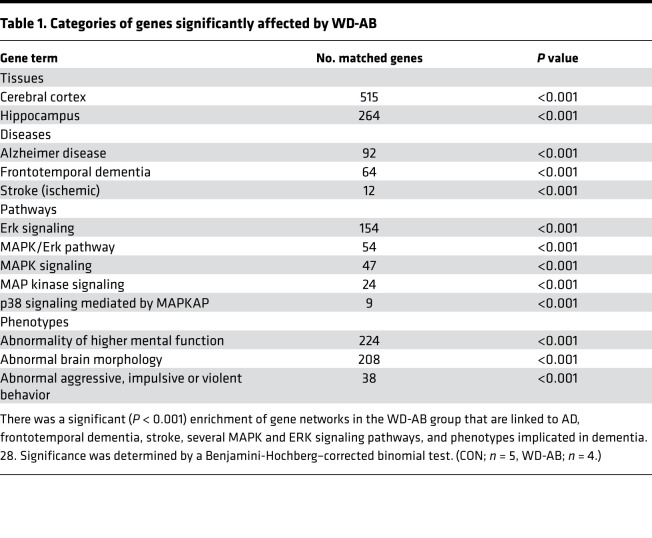
Categories of genes significantly affected by WD-AB

## Version 1 05/24/2021

Electronic publication
