# Supplementary material for: Cerebrovascular insufficiency and amyloidogenic signaling in Ossabaw swine with cardiometabolic heart failure
Source: JCI Insight. 2021 May 24;6(10):e143141. doi: 10.1172/jci.insight.143141 (PMC8262360; doi:10.1172/jci.insight.143141)
Supplement: Supplemental data [file jciinsight-6-143141-s071.pdf]

Supplement Material

Supplemental Methods

In vivo cardiovascular testing

Cardiovascular testing took place over two sessions as described previously.[1] In session one, swine were anesthetized using a Telazol/xylazine mixture (2.5 and 1.13 mg·kg^{-1}, respectively) and middle cerebral artery blood velocity was measured using transcranial Doppler ultrasound (Multigon). The Doppler signal was recorded using a Powerlab data acquisition system and extracted using Labchart 7 (Colorado Sprins, USA). In session two, swine were anesthetized using a Telazol/xylazine mixture (5 and 2.25 mg·kg^{-1}, respectively) and anesthesia was maintained using propofol (6-10 mg·kg·min^{-1}). The swine were placed in the supine position and HR (electrocardiogram; ECG), mean arterial pressure (fluid-filled 6F arterial catheter; Boston Scientific) and carotid artery blood flow (~2 cm inferior to the bifurcation; Doppler ultrasound) were recorded. Resting blood flow (velocity) and vascular resistance data were averaged over ~30 second periods. Vascular mechanics were averaged over 3 cardiac cycles and calculated as previously described.[1–3] After baseline measures were complete, vena cava occlusion was performed to elicit central hypovolemia.[1,3] Data were extracted before and during vena cava occlusion at 10 and 20 mmHg reductions in mean arterial pressure.[1,3] The Doppler audio signal was converted to an analogue signal using a custom Doppler audio translator[4] and all data were recorded using a Powerlab data acquisition system and extracted using Labchart 7. Body temperature was maintained using heating pads and blankets throughout the entire experiment.

Ex vivo arterial function

Cerebrovascular vasomotor function was examined as described previously.[1,5–7] Following euthanasia (exsanguination), 2a pial arteries and brain parenchymal arteries were transferred to a

Plexiglas chamber containing ice-cold physiological saline solution (PSS: NaCl 145 mM, KCl 4.7 mM, $CaCl_2$ 2.0 mM, $MgSO_4$ 1.17 mM with 10g/L albumin added; pH=7.4). The cerebral arteries were cannulated with two glass micropipettes (~80 µm in diameter) filled with PSS, warmed to 37°C and equilibrated at an intraluminal pressure of 60 mmHg for one hour. Vasomotor responses were examined under the following experimental conditions: 1) Exposure to sympathetic-co-transmitter, neuropeptide Y (NPY; $1e^{-9}$-$1e^{-6}$ M, half log doses). 2) Exposure to neurotransmitter, gamma amino butyric acid (GABA; $1e^{-10}$-$1e^{-3}$ M, whole log doses). 3) Exposure to neurotransmitter, acetylcholine (ACh; $1e^{-9}$-$1e^{-5}$ M, half log doses). 4) Exposure to nitric oxide synthase inhibitor, N-nitro-L-arginine methyl ester (L-NAME; $3e^{-4}$ M). Following completion of pharmacological experiments, vessels were washed twice with Ca^{2+} free PSS and exposed to $1e^{-4}$ M sodium nitroprusside (SNP) and the maximal lumen diameter was recorded. Percent vasoconstriction was calculated as the quotient of Δresponse and the maximal diameter in Ca^{2+} free PSS+SNP, multiplied by 100. Percent possible dilation was calculated as the quotient of Δ response and Δ maximal passive diameter in Ca^{2+} free PSS+SNP – baseline, multiplied by 100.[1,5,7] The area under or over the curve (relative to baseline; AUC) was calculated to display the net dilatory or constrictor response for each experimental condition.[8–10]

Immunoblots

Western blot experiments were performed as described previously.[11] Briefly, prefrontal cortical and hippocampal samples were homogenized (FastPrep®, MP Biomedicals, Santa Ana, CA) and protein concentration were determined using a Bicinchoninic acid assay (Sigma-Aldrich - B9643, VWR – BDH9312). The samples were prepared to contain equal concentrations (1µg/µl) of protein in 2x Laemmli buffer and placed in a dry bath at 100°C for 5 minutes. 20µg of protein were loaded and separated on 10% SDS-PAGE gels for 90 minutes at 120V. When analyzing C-

terminal fragment (CTF), the sample was separated on 16% Tris-tricine gels for 120 minutes at 120V. Proteins were then wet-transferred onto nitrocellulose membrane at 100V for 60 minutes. Membranes were blocked in Tris buffered saline/0.1% Tween 20 (TBST) with 5% non-fat powdered milk for 1 hour at room temperature. The appropriate primary antibody (1:500 ratio) was then applied and left to incubate on a shaker, at 4°C overnight. Following primary incubation, the membrane was washed with TBST 3 x 5 minutes and then incubated with the corresponding secondary antibody conjugated with horseradish peroxidase (Jackson ImmunoResearch, 1:2000 ratio) for 1 hour at room temperature. Signals were detected using enhanced chemiluminesence and were subsequently quantified by densitometry using a FluorChem HD imaging system (Alpha Innotech, Santa Clara, CA). Equal loading was confirmed with Ponceau staining.

Antibodies

Amyloid precursor protein (APP) C-Terminal Fragment (1:500, Biolegend, cat#SIG039152), beta-site amyloid precursor protein cleaving enzyme 1 (BACE1) (1:500, Cell Signaling cat#5606P), extra signal-regulated kinase (ERK) 1/2 (1:500, Cell Signaling cat#4695S), phosphorylated ERK (pERK) 1/2 (1:500, Cell Signaling cat#9101S), p38 (1:500, Cell Signaling cat #9212S), p-p38 (1:500, Cell Signaling cat #9211S), c-Jun N-terminal kinases (JNK) (1:500, Cell Signaling cat#9252S), pJNK (1:500, Cell Signaling cat #4671S), protein kinase B (AKT) (1:500, Cell Signaling cat #4685S), pAKT S473 (1:500, Cell Signaling cat #4058S), insulin degrading enzyme IDE (1:500, Santa Cruz cat#sc-393887).

Immunoprecipitation for markers of AD-related amyloidosis

The antibody targeting amino acids 676-695 (*e.g.* the C-terminal) of human APP695 (cat# A8717; Sigma-Aldrich) and the 6E10 antibody, which targets residues 1-16 of the amyloid beta (Ab) peptide (cat# SIG-39320; Cedarlane Laboratories Ltd.) were used to isolate and identify

various cleavage fragments of APP. Prefrontocortical and hippocampal samples (20-30 mg wet weight) were homogenized in 20 volumes of ice-cold RIPA buffer and centrifuged at 12,000x*g* (10 min; 4°C). 1250 μg of the supernatant, *e.g.*, the RIPA-*soluble* fraction, was first immunoprecipitated[12] for the full-length-APP using the C-terminally-directed antibody. This immunodepleted fraction was then immunoprecipitated using the 6E10 antibody (to isolate any Aβ peptide fragments). The anti-C-terminal immunocomplexes (C99 and C3-99) were resolved on 12% SDS-PAGE gels, whereas the 6E10 immunocomplexes were resolved on a discontinuous 8M urea gel system.[13] The fragments were detected using IRDye® 800CW Goat anti-Mouse IgG (H + L) [LICOR 925-32210] and densitometry was performed using supporting LICOR software.

Supplemental Results

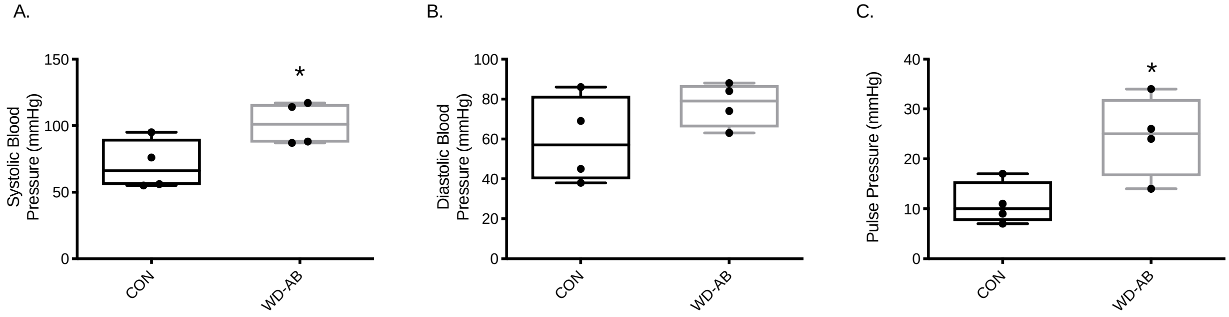

Supplemental Figure 1 Legend
Systolic (A), diastolic (b) and pressure (C) under anesthesia. Data analyzed using an unpaired, two-tail t-test. Values are represented as mean ± SEM. Significance indicated by (*) = $p < 0.05$ compared to control.

Supplemental Table 1:

Ingenuity Pathway and Gene Ontology Analyses of Induced Dementia/Behavioral-Related Gene Pathways Expressed between control and WD-AB in SG

Ingenuity Pathway Analysis		
**Tissues**		
**Name**	**Matched genes**	**P value**
Cerebral Cortex	BRCA2, Fah, Gbe1, Gch1, Gli3, Jag1, Maoa, OCRL, Pcca, PAFAH1B1, Notch3, Igf1, Agl, ALOX5, CSF3R, Grm3, GSTP1, GUCY1B1, RPS5, Adcy9, ADCYAP1R1, APBA1, Arg2, ATP6AP1, CALU, LDB2, DNAH8, Emp1, EXTL2, Frzb, Gabbr1, B4GALNT1, EIF3E, Acadsb, ARNT, ATP6V1B2, ATP6V1E1, BTN1A1, CTSC, CLU, COL4A2, COL6A2, CRYM, F3, FN1, GCLM, ITPR1, Jag2, Kcna6, KCNJ6, LAMB1, LAMB2, Limk1, LRP1, Lrp5, Mdk, MFAP2, Mst1r, Nfix, Pafah1b2, PAK1, PAWR, PCK1, Pde7a, Ppp2r2a, MAPK4, Nf2, Sfrp1, SNCG, SP2, SSR2, STXBP1, Tcf3, Thbs2, USP1, Fzd5, PIP4K2B, PPFIBP1, DCHS1, EIF3H, EIF4G3, Hap1, Map3k14, OSMR, AP2M1, Dctn1, EFEMP1, Fgf13, SREBF1, Traf4, VCAN, TRDMT1, Efnb1, ATP6V0D1, SYNGR1, B4galt6, Agtr1a, PLK1, Akap12, Hs3st1, APLNR, ATP6V0A1, Runx1t1, Elk1, GJA5, LIPE, Gjc1, Impa1, STMN1, Smad6, Nfib, Dll1, TP53BP1, PLIN3, Spry1, Mcm7, SNAI1, Wfs1, LANCL1, RAPGEF4, NSF, ENPP2, PENK, PPP5C, TFDP2, THY1, PROCR, CCT4, Paics, P3h4, CTCF, B3GNT2, DNAJB2, Mcm5, Ppp1r1a, LMO4, STIP1, LIAS, Fstl1, VCP, NUP50, DCTN3, PPA1,	<0.001

	PGP, RPL13, NCS1, PUM3, Slc35b4, ISYNA1, CCT5, DNM1L, TUSC3, ISCU, ATP6V1H, Cdipt, ARNT2, LRIG1, Irx3, Lhx6, MRAS, Plxna3, CAVIN1, UBR1, VCL, CACYBP, KIFAP3, MEIS2, Notch2, Per2, PLD3, PTPN13, SHC1, UBTF, Lynx1, MAP3K1, NCDN, Frat2, AHSA1, Cdc42ep4, LMOD1, DDAH1, Lamp5, EIF3K, St8sia5, HOOK2, Nme7, CLIC4, RGCC, Phpt1, Stxbp6, OLFM1, CADM1, GHITM, PCDH17, GOLIM4, CLSTN1, DNAJC6, Slitrk3, ZHX2, Zhx3, CMTR1, PPP1R13B, Nepro, Ppp1r16b, WSB1, LDLRAP1, Ripor2, MRPS7, EIF3L, PLCE1, HACD3, TMEM9, MRPL37, Syt3, Pard6a, Pole3, FERMT1, Sybu, P3H2, Foxj2, Actr10, CDCA7L, SYT6, FSTL5, PITHD1, CD248, DDX24, ZC3H8, Nrip3, Ubr4, Islr2, Fgf12, HNRNPH3, Kcnt1, DEF6, SH2D4A, NECAB1, Adcy5, COQ8A, Abcb6, SYNPR, MXRA8, ROGDI, SLTM, Chodl, CCDC92, TTC7A, NDFIP2, GLI2, Lrrc3, KLB, CCM2, Ccdc3, RAB34, PPP1R1B, Slitrk6, PDZD4, Card6, Cox4i2, B3GALT5, CCDC102A, Glce, TNKS1BP1, MADD, Cacng5, KHDRBS2, Grid2ip, SYT12, TMEM192, Lypd1, LRRC45, Cpt1c, PRICKLE1, FBXL16, Nyap1, Rnf149, GPR22, Plekhg1, CAMTA1, BEX4, Cacnb1, Chrm4, Glrb, Grm4, Oxtr, RARG, Bcat1, DPYSL3, CLCN3, CSPG4, Cd82, PFKP, PRKCG, PTPRA, YWHAB, Fzd1, PPFIA1, KAT2B, Dusp3, SLC1A3, SYT7, Acvr1b, Epha4, FXR2, ALDOC, MECOM, Rnd2, SLC1A5, ACTR1A, Stag1, Tmem50b, Zbtb18, FBLN1, HSPH1, Sox5, Ptpro, Sptlc2, UBA1, Ackr3, COL8A1,	

	KPNA6, Syt2, Cux1, Gabrg1, Slc22a3, MORF4L2, Tbk1, ABCA7, MTCH1, LMCD1, Stard8, ACIN1, TRIM2, CERCAM, DCXR, Echdc2, CA10, Rab37, EEF1D, ARL8A, ZNF275, Slc45a3, Acvr1c, SLC8A2, Lpcat4, Slc6a17, Fgd5, SNAI2, SRF, Lrp8, USP11, USP10, HNRNPH1, Sema3a, RIPK3, HNRNPUL1, RAD9A, SNX10, SV2A, RHBDD2, Tgif2, MRPS9, LRPPRC, CAMKK2, FOXP2, PHACTR1, DIDO1, COLEC12, IRF2BP2, Stum, ATP1B2, VDAC3, NPEPPS, MYO10, ACAA1, Acsl1, HMGB2, Hpcal1, ITGA7, Pgam1, MAPK1, PSMD8, TST, YWHAH, PPFIA3, COX5A, RPS6KA3, ABI1, PRCC, NCOR1, TAF9, MCM4, MTPN, ATXN10, Slc39a1, BRD1, NSFL1C, LMNA, ELOVL1, SMAP2, GRINA, NUDT7, P4HTM, Slc39a3, LRSAM1, ZC3H18, HUWE1, AP1G1, ATP2B4, Cdh4, Slc25a12, SYNJ1, Pfkfb4, Chaf1b, SCARB2, AKT2, EPB41L3, KAT6B, RBFOX2, PRKAA1, Rhou, FAM111A, EN1, Gprin3, ALDH9A1, Cat, Gsta4, DNAJA1, ARSD, KIF3C, Mapk3, TAF4, AP3B2, Mtmr6, ACTN4, PCMT1, GABBR2, COPS5, ACSS1, ATP2B2, Pgam2, ACSL4, PPP3CB, Ebf2, Ebf3, Six2, B3gat3, MAPRE3, ATP2C1, Rrm2b, RRP1B, NCOA7, CYP1B1, Entpd6, AP2B1, Ryk, CLGN, CREBBP, Rac3, SLC27A4, REEP5, Tubb4b, GABARAPL2, Grik1, NMT1, TUBA1C, ATP2A3, ANKRD11, DYNLRB1, VPS41, ATP9A, RBFOX1, CACTIN, Ggt7, Rasl11b, HSP90AB1, DPYSL2, Cdk2, IQGAP1, MAPRE2, RUSC1, Nmnat2, SPOCK1, Cpne6,	

	Ablim2, ZC3H4, RUFY3, RPS11, CMTM2, CYB5R1, Osbpl5, KIF1B, YWHAE, EEF1A1, TPM4, Them6, NPTN, YWHAQ, Ube4b, TMEM30A, Fry, WDR6, Aldh1l1, Bahcc1, MYH9, DCLK1, Nisch, ENO1, Mfap4, PPP2R5C, RRBP1, G3BP2, UBQLN1, DAZAP1, CALD1	
**Name**	**Matched genes**	**P value**
Hippocampus	OCRL, PAFAH1B1, Notch3, Igf1, ALOX5, GUCY1B1, RPS5, APBA1, CALU, LDB2, DNAH8, Emp1, EXTL2, Frzb, Gabbr1, B4GALNT1, EIF3E, ARNT, Atp6v1b2, Calcr, Clu, COL6A2, Crym, Foxo1, Fn1, ITPR1, Jag2, KCNJ6, LAMB1, Limk1, LRP1, Mdk, MFAP2, Mst1r, PAK1, PAWR, PCK1, Ppp2r2a, MAPK4, Sfrp1, SNCG, SP2, Tcf3, Thbs2, USP1, Fzd5, DCHS1, Eif3h, EIF4G3, Hap1, OSMR, Dctn1, EYA4, Fgf13, Traf4, Vcan, TRDMT1, Efnb1, Agtr1a, Akap12, APLNR, GJA5, Gjc1, STMN1, Smad6, Dll1, TP53BP1, PLIN3, Spry1, SNAI1, Wfs1, NFE2, NSF, Enpp2, PENK, PPP5C, TFDP2, Mcm5, Ppp1r1a, Lmo4, LIAS, VCP, NUP50, PPA1, PGP, NCS1, PUM3, CCT5, DNM1L, ATP6V1H, Lrig1, Lhx6, CACYBP, KIFAP3, MEIS2, Notch2, Per2, PLD3, PTPN13, UBTF, Lynx1, MAP3K1, NCDN, AHSA1, Cdc42ep4, Lamp5, St8sia5, HOOK2, Olfm1, GOLIM4, Mfn2, Slitrk3, CMTR1, PPP1R13B, RAB11FIP5, Ppp1r16b, Ripor2, MRPS7, PLCE1, MRPL37, Syt3, Pard6a, P3H2, SYT6, FSTL5, ZC3H8, Nrip3, Islr2, NECAB1, Abcb6, MXRA8, SLTM, GLI2, KLB, CCM2, Ccdc3, Ppp1r1b, Slitrk6, Card6, CCDC102A,	<0.001

	Abhd3, Glce, Cacng5, KHDRBS2, Grid2ip, SYT12, TMEM192, Lypd1, LRRC45, Plekhg1, Cacnb1, Chrm4, Glrb, Grm4, Oxtr, RARG, CSPG4, Cd82, PRKCG, YWHAB, PPFIA1, Dusp3, Slc1a3, Syt7, Epha4, FXR2, ALDOC, MECOM, Rnd2, Zbtb18, HSPH1, Ackr3, KPNA6, Syt2, Gabrg1, Slc22a3, ABCA7, MTCH1, ACIN1, TRIM2, Rab37, Acvr1c, Slc8a2, USP10, HNRNPH1, Sema3a, RIPK3, HNRNPUL1, Sv2a, MRPS9, LRPPRC, Camkk2, FOXP2, PHACTR1, Stum, VDAC3, MYO10, ACAA1, Hpcal1, ITGA7, MAPK1, PSMD8, Ywhah, PPFIA3, COX5A, ABI1, NCOR1, TAF9, MCM4, LMNA, P4HTM, ZC3H18, HUWE1, AP1G1, Cdh4, Slc25a12, SCARB2, AKT2, KAT6B, RBFOX2, PRKAA1, Rhou, EN1, Gprin3, ARSD, AP3B2, COPS5, ATP2B2, ACSL4, Ebf2, Ebf3, MAPRE3, ATP2C1, RRP1B, CLGN, Rac3, SLC27A4, Tubb4b, GABARAPL2, NMT1, ATP2A3, ANKRD11, Rasl11b, DPYSL2, MAPRE2, Cpne6, Ablim2, ZC3H4, RUFY3, CMTM2, CYB5R1, YWHAE, EEF1A1, TPM4, Them6, Aldh1l1, Bahcc1, DCLK1, Nisch, Eno1, Mfap4, RRBP1, G3BP2, DAZAP1	
**Diseases**		
**Name**	**Matched genes**	**P value**
Alzheimer Disease	ABCA7, ACVR1B, ADAM22, ADAMTS4, AGRN, AHNAK, AKT2, ALAS2, ALOX5, APBA1, APBB3, AZU1, BTN1A1, C1R, CAMKK2, CAPRIN2, CAT, CLSTN1, CLU, COLEC12, COX5A, CSF1, CSPG4, DENND5B, DKK2, DNM1L, DOCK6,	<0.001

	DPYSL2, DYNC1H1, EMP1, FFAR4, GCG, GPR137, GRM5, GSK3B, GUCY1B1, HACD3, HIC1, IQUB, ITGA11, ITPR1, KIDINS220, LAMA4, LIMK1, LMBRD2, LRP1, LRP5, LRP6, LRP8, MAOA, MAPK1, MAPK3, MAPK9, MPO, MSR1, MTOR, NPEPPS, NT5E, P2RX6, PAK1, PANX2, PCMT1, PER2, PLCE1, PLD3, PPARG, PPARGC1B, PPEF1, PPID, PPP3R1, PREX2, PRND, RAB13, RALGPS2, SEMA3A, SERTAD4, SLC18A3, SLC1A3, SLC25A4, SLC39A1, SNCG, SPTLC2, TGFB3, TM2D3, TNFRSF21, TONSL, TUBA1C, TXNRD3, UBQLN1, UNC5C, VCP, VSTM4	
Frontotemporal Dementia	AAK1, AP3B2, APLNR, ATP2B2, ATXN10, CABP1, CALD1, CAMTA1, CAVIN1, CRYM, CUX2, DAB2, DCLK1, DCTN1, ELOVL4, FGF13, FMO2, FOXD1, FRZB, GABBR2, GLI3, GLRB, GOLIM4, GPR22, GPRASP1, GRM5, GRM7, HNRNPH1, ISYNA1, ITPR2, KCNJ6, LAMP5, LDB2, LRRC8B, MADD, MAPRE3, MYOT, MYT1L, NAP1L2, NAP1L3, NCDN, NET1, NMNAT2, NPTXR, NRIP3, NSF, OLFM1, OMD, PHYHIP, PPP3R1, RAB39B, RBFOX1, RBP4, SLC8A2, SNX10, SPG11, STXBP1, TBK1, THY1, TRHDE, UBQLN1, VCAN, VCP, XK	<0.001
Stroke (Ischemic)	ACE2, ACSL4, AGTR1, ALOX5, F3, HSPA4, IQGAP1, MAPK3, NOTCH3, PECAM1, PROCR, TSPAN33	<0.001
**Pathways**		
**Name**	**Matched genes**	**P value**

Erk Signaling	ACTN4, AKT3, RALB, ROCK1, RARA, EPHB4, ADCY5, COL8A1, CACNG2, MYO1C, IQGAP1, FLT3, PAK3, PPP1R1A, PRKAA2, LAMA4, BMP2, COL27A1, COL2A1, COL6A2, RARG, GSK3B, CACNA2D2, PPP2R5C, CSF1, PPP1R1B, PRKCG, CACNG5, ELK1, MAP3K11, MMRN2, KRT18, ARHGEF15, PPP2R5B, NCF2, SOX18, DCHS1, SOX5, LRP5, ITGA7, ANGPTL1, FGF22, PPP1R3C, IL2RB, ELK4, ARAF, SRF, CDC25B, LIF, ARHGEF2, PLCE1, MYH9, MYLK, MRAS, STMN1, DDR2, PPP1R13B, PRKAA1, MST1R, SMAD4, PARVA, AKT2, FZD5, E2F2, LAMC2, FGF13, PPP1R3B, GMFG, CDC42EP4, CDC42EP5, COL4A4, PENK, RYK, RHOD, RALA, UBTF, TGFB3, NOTCH3, LAMB1, ADCY9, TNFRSF21, CSF3R, NPTN, FN1, GNA14, TEK, PPP1R10, ELN, PPP2R2A, CDH4, VCAN, GNG11, VCL, VEGFB, SPOCK1, FGF12, TCF3, RAC2, NBN, TIE1, DUSP3, TGFB2, PIP5K1B, CD247, RND2, LAMB2, MCF2L, ALK, IGF1, ECM1, EFEMP1, LAMC3, FOXO1, CREBBP, MAPK9, CDC42EP1, CDH12, PARD6A, LIMK1, LRP6, PAK1, NET1, PIP4K2C, ITGA11, RAC3, SEMA3A, MAPK11, CLEC11A, FZD1, COL4A2, RPS6KA3, MTOR, CACNA2D1, CD4, ITGA8, COL15A1, NOTCH2, FZD9, LIMS2, LTBP1, LAMA5, OSMR, CDK2, PIP4K2B, CACNB1, MYO10, ETS2, EPHA4, MDK, MAPKAPK2, SNAI1, SNAI2, MAPK3, MAPK1	<0.001

MAPK ERK Pathway	EPHB4, PPARGC1A, CAMK1D, PRKAA2, YWHAH, GSK3B, PRKCG, ELK1, MAP3K1, PPARGC1B, YWHAQ, YWHAB, DAPK2, ITGA7, MITF, PPARD, SRF, CDC25B, ARHGEF2, FSCN2, PPP3R1, PRKAA1, FZD5, RGS7BP, UBTF, NFKB2, CREB3L2, MYT1L, PPARG, GNA14, YWHAE, SHC1, VAV2, RAC2, SHC2, IMPA1, EMB, LIMK1, TMOD4, PAK1, ITGA11, RAC3, FZD1, RAPGEF3, HSP90AB1, ITGA8, TONSL, FZD9, ADAM12, ETS2, EPHA4, MAPKAPK2, MAPK3, MAPK1	<0.001
MAP Kinase Signaling	YWHAH, ELK1, MAP3K11, YWHAQ, YWHAB, ARAF, SRF, TAB3, MRAS, SPRY1, RASGRP3, PPP2R2A, YWHAE, RAC2, DUSP4, DUSP3, GSTP1, MAPK9, RAC3, MAPK11, RPS6KA3, MAPKAPK2, MAPK3, MAPK1	<0.001
MAPK Signaling Pathway	AKT3, CACNG2, FLT3, CACNA2D2, CSF1, PRKCG, CACNG5, ELK1, MAP3K1, MAP3K11, STK3, FGF22, ELK4, ARAF, SRF, CDC25B, MRAS, STMN1, PPP3R1, PPP3CB, ACVR1C, AKT2, NFKB2, TGFB3, RASGRP3, TEK, PPP5C, VEGFB, RAC2, DUSP4, DUSP3, TGFB2, IGF1, MAPK9, PAK1,	<0.001
P38 Signaling Mediated By MAPKAP Kinases	YWHAH, YWHAQ, YWHAB, SRF, CDC25B, YWHAE, TCF3, LSP1, MAPKAPK2	<0.001
**Phenotypes**		
**Name**	**Matched genes**	**P value**
Abnormality of Higher Mental Function	AKT3, ADAM22, PNPLA2, ADCY5, CACNG2, THOC2, DNAJC6, MAPRE2,	<0.001

	SPRY4, DCTN1, PAK3, PIGP, NAB2, ZBTB18, POMGNT2, MFN2, PNPLA6, AGL, COL2A1, FBLN1, COQ8A, CYB5A, CACNA2D2, CEP19, PRKCG, KIF7, ELMO2, COLEC11, MMP14, ALOX12B, KRT18, AP3B2, CUX2, STAG1, CUX1, XK, DCHS1, SKI, POLA1, WFS1, MLXIPL, AP2M1, SOX5, PRICKLE1, GABBR2, LRP5, ITGA7, SLC25A4, IL2RB, MPO, AMT, SPG11, ALX3, ABCA7, SLC25A12, ANK1, NFIX, GAN, SLC12A6, MYOT, ARHGEF2, SCARB2, ABHD12, MRAS, SLC1A3, SLC5A7, SLC6A17, CAVIN1, NBAS, FSCN2, SLITRK6, OCRL, LMBR1, HACE1, STX1B, STXBP1, MTFMT, SMAD4, ADAMTS3, GCH1, GHR, AKT2, LMNA, DNAJB2, ARNT2, NDE1, UBE2A, TTBK2, GJA5, JAG1, UBA1, DLL1, DMXL2, REEP2, ATP6AP1, HUWE1, AGRN, RRM2B, SAMD12, AR, ATP6V1B2, SALL1, ZSWIM6, B4GALNT1, FRRS1L, ITPR1, KAT6A, NDUFS1, SACS, CDK13, SEMA3D, TBK1, KCNJ6, TBX1, GLI2, UBTF, NECAP1, NFKB2, GLRB, AHI1, NOTCH3, GLI3, LAMB1, DOCK6, P4HTM, ADGRV1, PAFAH1B1, CAMTA1, HERC1, NALCN, TUSC3, BRCA2, ATP13A2, MEIS2, ELOVL4, WDR11, DNAJC13, MYT1L, VCP, DNM1L, PPARG, EFNB1, SYT2, HECW2, SEC23B, FOXP2, UBR1, USP9X, ELN, ELOVL1, PLD3, NF2, TWNK, SEC61A1, EBF3, CAMKMT, DSTYK, GBE1, ROGDI, FGF12, PSMD12, MAB21L2, DYNC1H1, PIGA, NBN, IMPA1, MAOA, SLC18A3, CD247, ANKRD11, LAMB2,	

	RAB27A, IGF1, ECM1, LAS1L, EHMT1, DNAJC5, SDR9C7, CREBBP, LIMK1, PGAP1, MASP1, ACSL4, PAK1, OPA3, CDC45, KAT6B, KANK1, KCTD17, FOLR1, RAB39B, KCNJ8, KCNN3, CCM2, CISD2, RAI1, SEMA3A, KIDINS220, PCCA, KIF1B, BNC2, RPS6KA3, GATA6, ACADSB, KCTD7, NUS1, KIZ, FGFRL1, ATP6V1E1, MTOR, KCNT1, CNNM2, TONSL, ATXN10, CTCF, TPM2, NTN1, SYNJ1, NCAPG2, EMC1, YAP1, MAST1, SNAI2, MAPK1	
Abnormal Brain Morphology	AKT3, PNPLA2, EPHB4, THOC2, PPP2R3C, DNAJC6, MAPRE2, SPRY4, PAK3, PIGP, ZBTB18, POMGNT2, BMP2, MFN2, PNPLA6, COL2A1, FBLN1, COQ8A, CYB5A, CACNA2D2, PRKCG, KIF7, LIAS, MAP3K1, CHST3, AP3B2, CUX2, STAG1, CTSC, DCHS1, SKI, POLA1, WFS1, MLXIPL, PCK1, SOX5, B3GAT3, GABBR2, LRP5, SLC25A4, MPO, AMT, SPG11, ALX3, ABCA7, MITF, ANK1, NFIX, GAN, SLC12A6, ARHGEF2, MYLK, BMPER, SCARB2, ABHD12, SLC1A3, SLC6A9, SLC25A46, DDR2, NAT8L, CAVIN1, PHACTR1, OCRL, LMBR1, HACE1, STX1B, STXBP1, MTFMT, SMAD4, ADAMTS3, G6PC3, GCH1, LMNA, ARNT2, NDE1, UBE2A, TTBK2, GJA5, JAG1, DLL1, DMXL2, PROS1, HUWE1, RRM2B, AR, SALL1, ZSWIM6, B4GALNT1, FRRS1L, AGTR1, ITPR1, KAT6A, LDLRAP1, NDUFS1, SACS, CDK13, TBK1, KCNJ6, TBX15, BGN, TBX1, GLI2, UBTF, NECAP1, NFKB2, AHI1, TGFB3, NOTCH3, GLI3, LAMB1, SNX10, DOCK6, P4HTM,	<0.001

	ADGRV1, PAFAH1B1, CAMTA1, HERC1, NALCN, TUSC3, BRCA2, ATP13A2, PRUNE1, MEIS2, ELOVL4, WDR11, DNAJC13, VCP, DNM1L, PPARG, EFNB1, HIC1, HECW2, SEC23B, FN1, FOXP2, UBR1, USP9X, ELN, PLD3, YWHAE, NF2, TWNK, EBF3, DSTYK, GBE1, ROGDI, FGF12, PSMD12, DYNC1H1, PIGA, NBN, FAM111A, SLC18A3, TGFB2, ANKRD11, RAB27A, MCM4, IGF1, ECM1, LAMC3, LAS1L, EHMT1, DNAJC5, CREBBP, LIMK1, PGAP1, MASP1, ACSL4, CHP1, PAK1, OPA3, CDC45, KAT6B, KANK1, RAB39B, CCM2, CISD2, RAI1, SEMA3A, PROC, CRELD1, KIDINS220, PCCA, KIF1B, COL4A2, RPS6KA3, GATA6, ACADSB, KCTD7, NUS1, FGFRL1, ATP6V1E1, MTOR, KCNT1, CNNM2, NOTCH2, TONSL, ATXN10, CTCF, NTN1, SYNJ1, AGTPBP1, NCAPG2, EMC1, MCM5, MAST1, SNAI2, MAPK1	
Abnormal Aggressive, Impulsive or Violent Behavior	THOC2, PAK3, PIGP, EFHC1, CUX2, DCHS1, SKI, AP2M1, SOX5, GABBR2, AMT, NFIX, SLC6A17, NAT8L, OCRL, STXBP1, NDE1, UBE2A, NFIB, TBX1, UBTF, CAMTA1, NALCN, ATP13A2, MYT1L, VCP, HECW2, USP9X, PSMD12, IMPA1, MAOA, ECM1, EHMT1, CREBBP, RAI1, RPS6KA3, KCNT1, ATXN10	<0.001

References

1. Olver TD, Hiemstra JA, Edwards JC, et al. The loss of female sex hormones exacerbates cerebrovascular and cognitive dysfunction in aortic banded mini-swine through a NPY-BKCa-NO mediated mechanism. *JAHA*. 2017;6(11):pii: e007409.
2. O'Rourke MF, Staessen JA, Vlachopoulos C, Duprez D, Plante GE. Clinical applications of arterial stiffness; definitions and reference values. *Am J Hypertens*. 2002;15(5):426-444.
3. Olver TD, Klakotskaia D, Ferguson BS, et al. Carotid artery vascular mechanics serve as biomarkers of cognitive dysfunction in aortic-banded miniature swine that can be treated with an exercise intervention. *J Am Heart Assoc*. 2016;5(5):e003248.
4. Herr MD, Hogeman CS, Koch DW, Krishnan A, Momen A, Leuenberger UA. A real-time device for converting Doppler ultrasound audio signals into fluid flow velocity. *Am J Physiol Hear Circ Physiol*. 2010;298:1626-1632. doi:10.1152/ajpheart.00713.2009.
5. Olver TD, McDonald MW, Klakotskaia D, et al. A chronic physical activity treatment in obese rats normalizes the contributions of ET-1 and NO to insulin-mediated posterior cerebral artery vasodilation. *J Appl Physiol (1985)*. 2017;122(4):1040-1050. doi:10.1152/japplphysiol.00811.2016
6. Olver TD, Edwrads JC, Jurrissen TJ, et al. Western diet-fed, aortic-banded Ossabaw swine: A preclinical model of cardio-metabolci heart failure. *JACC Basic to Transl Sci*. 2019;4(3):404-421. doi:10.1016/j.jacbts.2019.02.004
7. Olver TD, Grunewald ZI, Jurrissen TJ, et al. Microvascular insulin resistance in skeletal muscle and brain occurs early in the development of juvenile obesity in pigs. *Am J Physiol Integr Comp Physiol*. 2018;314(2):R252-R264.

8. Olver TD, McDonald MW, Grisé KN, et al. Exercise training enhances insulin-stimulated nerve arterial vasodilation in rats with insulin-treated experimental diabetes. *Am J Physiol Regul Integr Comp Physiol*. 2014;306(12):R941-R950.

9. Inocencio IM, Polglase GR, Miller SL, et al. Effects of maternal sildenafil treatment on vascular function in growth-restricted fetal sheep. *Arterioscler Thromb Vasc Biol*. 2019;39:1-10.

10. Olver TD, Grunewald ZI, Ghiarone T, et al. Persistent insulin signaling coupled with restricted PI3K activation causes insulin-induced vasoconstriction. *Am J Physiol Hear Circ Physiol*. 2019;317(5):1166-1172.

11. Hayward GC, Leblanc PJ, Emter CA, et al. Female sex hormones and cardiac pressure overload independently contribute to the cardiogenic dementia profile in Yucatan miniature swine. *Front Cardiovasc Med*. 2019;6(129):1-13. doi:10.3389/fcvm.2019.00129

12. Quartey MO, Nyarko JNK, Pennington PR, et al. Age- and sex-dependent profiles of APP fragments and key secretases align with changes in despair-like behavior and cognition in young APPSwe/Ind mice. *Biochem Biophys Res Commun*. 2019;511(2):454-459. doi:10.1016/j.bbrc.2019.02.083

13. Nyarko JNK, Quartey MO, Pennington PR, et al. Profiles of β-Amyloid Peptides and Key Secretases in Brain Autopsy Samples Differ with Sex and APOE ε4 Status: Impact for Risk and Progression of Alzheimer Disease. *Neuroscience*. 2018;373:20-36. doi:10.1016/j.neuroscience.2018.01.005

14. Lee J, Gao C, Peng G, et al. New Methods in Cardiovascular Biology Analysis of Transcriptome Complexity Through RNA Sequencing in Normal and Failing Murine Hearts HF. 2011:1332-1341. doi:10.1161/CIRCRESAHA.111.249433

15. Gao C, Chen J, Wang Y, et al. and heart failure RBFox1-mediated RNA splicing regulates cardiac hypertrophy and heart failure. 2016;126(1):195-206. doi:10.1172/JCI84015.lator

16. Gao C, Howard-quijano K, Rau C, et al. Inflammatory and apoptotic remodeling in autonomic nervous system following myocardial infarction. 2017:1-17.

17. Patro R, Duggal G, Love MI, Irizarry RA, Kingsford C. Salmon provides fast and bias-aware quantification of transcript expression. *Nat Methods*. 2017;14(4):417-419. doi:10.1038/nmeth.4197

18. VQ V. ggbiplot: A ggplot2 based biplot. R package version 0.55. http://github.com/vqv/ggbiplot.

19. Langfelder P, Horvath S. WGCNA : an R package for weighted correlation network analysis. 2008. doi:10.1186/1471-2105-9-559

20. Fuchs SB, Lieder I, Stelzer G, Mazor Y, Buzhor E. Original Articles. 2016;20(3):139-151. doi:10.1089/omi.2015.0168

21. Toedebusch RG, Roberts MD, Wells KD, et al. Unique transcriptomic signature of omental adipose tissue in Ossabaw swine: a model of childhood obesity. *Physiol Genomics*. 2014;46(10):362-375. doi:10.1152/physiolgenomics.00172.2013

22. Padilla J, Jenkins NT, Lee S, et al. Vascular transcriptional alterations produced by juvenile obesity in Ossabaw swine. *Physiol Genomics*. 2013;45(11):434-446. doi:10.1152/physiolgenomics.00038.2013
